# Risk Factors of the First Febrile Seizures in Iranian Children

**DOI:** 10.1155/2010/862897

**Published:** 2010-06-24

**Authors:** Abolfazl Mahyar, Parviz Ayazi, Mazdak Fallahi, Amir Javadi

**Affiliations:** ^1^Department of Pediatrics, Quds Children Hospital, Qazvin University of Medical Sciences, Quds Square, Qazvin 34159-1-4595, Iran; ^2^Department of Social Sciences, Qazvin University of Medical Sciences, Qazvin 34159-1-4595, Iran

## Abstract

*Objective*. Febrile seizures are the most common type of convulsion in children. The identification of influencing factors on incidence of the first febrile seizures is of prime priority. The aim of this study was to identify the risk factors of the first febrile seizures in Iranian children. *Methods*. In this case-control study 80 children aged 9 month to 5 years with their first febrile seizures were compared with 80 children with fever without seizure based on different risk factors in 2007. *Results*. There was significant difference between two groups regarding the gender, family history of febrile seizures, breast-feeding duration, and the body temperature (*P* < .05). *Conclusion*. Our study showed that factors including the gender, family history of febrile seizures, breast-feeding duration, and the body temperature are among the risk factors in occurrence of the first febrile seizure. Preventive measures to remove such risk factors could lead to lower the incidence of febrile seizures.

## 1. Introduction

Convulsion is the most common neurologic finding in children as 10% of kids experience such clinical condition sometime during their life. There are several types of convulsion, but the most common type of the disease is the febrile seizures observed as simple and complex febrile seizures [1]. The prevalence of febrile seizures in children is approximately 3%-4% and usually develops in children of 9 months to 5 years old following an increase in body temperature higher than 39°C [2]. There are several reports indicating that different factors including the environmental and genetic factors influencing the incidence of febrile seizure [1, 2]. In some studies it has been emphasized that the mothers' disease during pregnancy, prematurity, and delivery complications could be considered as risk factors for occurrence of the first febrile seizures [3]. 

Although the bulk of researches carried out over the last 25 years is indicative of a good prognosis regarding most of the cases of febrile seizures, nevertheless, there are also reports in which the risk of developing epilepsy to more than 9% in presence of risk factors in these patients is highlighted [2, 4].

 Regarding the high prevalence of febrile seizures in children, efforts have to be made in identifying the influential risk factors associated with the occurrence of disease; hence, the present study to investigate the risk factors of febrile seizures in children was attempted in 2007.

## 2. Materials and Methods

In this case-control study, 80 children aged from 9 months to 5 years with their first febrile seizures (case group) were compared with 80 children with fever without seizure (control group) based on febrile seizures-associated risk factors in 2007. Both groups were matched according to age. Every member of the case group showed all the classic criteria of febrile seizure experiencing their first febrile seizures. The members of the control group were febrile but without seizure taken to the pediatric clinic due to ordinary illness such as common cold. The body temperature was measured using standard protocol for axillary body temperature measurement. Following full explanation of the project for parents, signed written agreements were obtained. A questionnaire was completed by every single parent followed by statistical analysis of data using SPSS software and chi square test. A *P* value <.05 was considered as significant.

## 3. Results

Of 80 children with febrile seizures 53 cases (66%) were male and 27 (34%) females whereas in control group the similar figures were 36 cases (45%) and 44 (55%) for males and females, respectively. Statistically, a significant difference was found between two groups regarding the gender (*P* = .01).

The minimum age in children with febrile seizures (case group) was 5 months and the maximum 58 months and a mean of 23.38 ± 12.14 months. Similarly, the minimum, maximum, and mean age among the control group was 10, 60, and 26.58 ± 12.57, respectively. There was no significant difference between two groups regarding the age (*P* > .05).

Among the case group 64 cases (80%) were breast-fed and 16 children (20%) bottle-fed. Similarly, Breast-feeding and bottle-feeding were seen in 71 cases (88.8%) and 9 (11.2%) of control group, respectively, (*P* > .05). The mean breast-feeding duration was 15.38 ± 6.88 months in case group and 19 ± 5.35 in control group with a significant difference between two groups, statistically (*P* = .000).

The most common types of diseases in case group were upper respiratory tract infections in 42 children (53.8%), gastroenteritis in 19 cases (24.4%), acute otitis media in 7 subjects (9%), urinary tract infection in 5 cases (6.4%), and pneumonia in 3 patients (3.8%), respectively. The variety of diseases observed in control group were gastroenteritis in 28 cases (35%), viral infections of upper respiratory tract in 21 children (26.2%), pneumonia in 8 subjects (10%), urinary tract infection in 7 patients (8.8%), and acute otitis media in 5 cases (6.3%), respectively. No significant difference associated with the background diseases between two groups was established (*P* > .05).

The mean body temperature in case and control groups was 38.9 ± 0.37 and 38.3 ± 0.43°C, respectively. Regarding the body temperature, a significant difference was observed between two groups (*P* = .000). The mean birth weight in case group was 3180.87 ± 479.06 gr and 3164.37 ± 284.66 in control group without a significant difference between two groups, statistically (*P* > .05). concerning the history of febrile seizures, 44 cases (55%) of case group and 5 patients (6.3%) in control group were found to have a history of such clinical complication (*P* < .000). Pre-maturity was observed in 6 cases (7.6%) of case group and 5 children (6.3%) in control group with insignificant difference between two groups (*P* > .05). Vaginal labor was found in 43 cases (53.8%) of case group and 36 subjects (45%) of control group whereas cesarean section was demonstrated in 37 (46.3%) and 44 (55%) of the case and control groups, respectively, (*P* > .05). No labor with forceps or vacuum was observed in case group, however, vacuumed delivery was found in one case (1.3%) of control group (*P* > .05). Low apgar score was found in 7 children (8.8%) of case group and 2 cases (2.5%) of control group who needed resuscitation measures at birth time whereas other cases in both groups showed normal apgar scores (*P* > .05). Smoking was observed in 2 patients (2.5%) of case group with no counterpart in control group (*P* > .05). Coffee consumption was demonstrated in 3 subjects (3.8%) of case groups and 2 children (2.5%) of control group with insignificant difference between two groups (*P* > .05). A history of disease in mother during pregnancy was observed in 6 cases (7.5%) of case group and 9 cases of control group. The most common diseases in mothers were PROM, preeclampsia, hypertension, and bell's paralysis as shown in Table 1 (*P* > .05).

## 4. Discussion

The present study revealed that the gender, family history of febrile seizures, breast-feeding duration, and high body temperature are among the risk factors associated with the first febrile seizures in children. Febrile seizures are the most common type of convulsion in children diagnosed in 3%-4% of children worldwide. Although the exact cause of febrile seizure is unknown, however, there are several factors considered as risk factors associated with incidence of the first febrile seizures. Ellatiff reported that the upper respiratory infections, family history of febrile seizure, prematurity, and labor difficulties are the major risk factors of the first febrile seizures [5]. Berg gave a description, in his paper, of gastroenteritis and smoking during pregnancy as important risk factor for the first febrile seizure [6]. Talebian emphasized on family history of convulsion [7] andHuangmentioned developmental disorder as risk factors of the first febrile seizure [8]. In a study by Gohnston, the author highlighted the genetic role for the occurrence of disease and that the illness is an autosomal disease [2]. In other studies it was revealed that the prematurity and labor difficulties [3] and prenatal asphyxia [9] to be considered as important risk factors of febrile seizures. In the past, there were some considerations for viruses playing a role in appearance of febrile seizures, nevertheless, a study by Chung showed that there was no discrepancy in role of Influenza viruses, Parainfluenza viruses, and Adenoviruses and that the risk of febrile seizures is lower following the rotaviral infections [10]. No reference to body temperature equal or higher than 39°C as a risk factor was made in the literature [10] which is inconsistent with the data found in our study. There is little data concerning the preventive effect of breast-feeding on occurrence of febrile seizures. Farivar reported that breast-feeding confers a preventive effect against the incidence of febrile seizures [11]. It was also shown in our study that the mean breast-feeding duration in children with their first febrile seizures was significantly lower than those of healthy children. In our study, although no significant correlation regarding the background diseases was established between two groups, yet it was demonstrated that the upper respiratory infections are among the common causes of disease. Having considered the data of different studies mentioned earlier, it is clear that a variety of different factors could be regarded as risk factors of febrile seizures, thus; identification of such predisposing factors in each community should be looked upon as a prime medical necessity. Although the febrile seizures in most cases is accompanied with a good prognosis, however in presence of different risk factors such as family history, the risk of developing epilepsy increases to almost 9% [1, 2, 12]. Hence, attempts have to be made to accurately identify these risk factors and eliminate them, if possible.

## 5. Conclusion

Based on our data, it was revealed that the parameters such as gender, family history of febrile seizures, breast-feeding duration, and body temperature are among the risk factors in occurrence of the first febrile seizure and, thus, preventive measures in removing these risk factors could lead to a decrease in incidence of febrile seizures.

## Figures and Tables

**Table 1:** Comparison of Risk Factors among children with first febrile Seizures (case group) and healthy children (control group) in Qazvin, Iran.

Risk factor	Case	Control	*P*
Gender	M: 66% F: 34%	M: 45% F: 55%	.01
Family history of FS	55%	6.3%	.000
Breastfeeding duration (Mean)	15.38 ± 6.88 months	19 ± 5.35 months	.000
Temperature (Mean)	38.9 ± 0.37°C	38.4 ± 0.43°C	.000
Family history of Epilepsy	7.5%	5.1%	>.05
Breast Feeding	80%	88.8%	>.05
Background Diseases	UTI, GE, AOM, others	GE, URI, Pneumonia, others	>.05
Birth Weight (Mean) gr	3180.84 ± 479.06	3164.37 ± 284.66	>.05
Prematurity	7.6%	3.8%	>.05
Cesarean labor	46.3%	55%	>.05
Labor with Vacuum, Forceps	0%	1.3%	>.05
Low Apgar and Resuscitation	8.8%	2.5%	>.05
Maternal Smoking	2.5%	0%	>.05
Coffee consumption	3.8%	2.5%	>.05
Maternal disease	7.5%	11.2%	>.05
